# Field‐deployable, ultrasensitive and visual detection systems targeting severe fever with thrombocytopenia syndrome virus (*Dabie bandavirus*) based on CRISPR/Cas12a and DNAzyme

**DOI:** 10.1002/ctm2.70393

**Published:** 2025-07-03

**Authors:** Junhu Wang, Ming Yue, Yingqing Mao, Qiong Chen, Yixin Ge, Haiming Yi, Hongming Wang, Sunjie Yang, Jin Zhu, Yuexi Li, Yong Qi

**Affiliations:** ^1^ Huadong Research Institute for Medicine and Biotechniques Nanjing Jiangsu Province China; ^2^ Infectious Diseases Department The First Affiliated Hospital of Nanjing Medical University Nanjing China; ^3^ School of Engineering China Pharmaceutical University Nanjing China; ^4^ School of Life Science Xuzhou Medical University Xuzhou China

1

Dear Editor,

We present this letter to highlight the development of two innovative CRISPR‐based visual detection systems (RAA‐CRISPR/Cas12a‐fluorescent [RCCF] and RAA‐CRISPR/Cas12a‐DNAzyme [RCCD]) for severe fever with thrombocytopenia syndrome virus (SFTSV). The detection systems address critical limitations in field and laboratory testing. Our work demonstrates ultrasensitive and specific detection of SFTSV RNA using recombinase‐aided amplification (RAA) combined with CRISPR/Cas12a and different reporters. These methods enable rapid, cost‐effective and naked‐eye visual detection, with distinct advantages for different scenarios: RCCF suits laboratories/lab‐based point‐of‐care testing (POCT) via fluorescence, whereas RCCD is ideal for resource‐limited settings using colourimetry.

Severe fever with thrombocytopenia syndrome (SFTS), a life‐threatening illness, is induced by the *Dabie bandavirus*, which is also known as SFTSV and is mainly transmitted through ticks.^1^ The fatality rates of patients with SFTS can reach up to 30%.^2^ In 2018, the WHO designated SFTSV infection as a prioritized disease for research and development in emergency contexts.^3^ Currently, the main techniques for detecting SFTSV include enzyme‐linked immunosorbent assay (ELISA), virus isolation and cultivation and reverse transcription‐polymerase chain reaction (RT‐PCR). However, ELISA has relatively low sensitivity and specificity, virus isolation and culture are time‐consuming and RT‐PCR involves higher costs and longer processing times.^4^ Consequently, none of these methods are suitable for field detection. Rapid, sensitive and specific detection of SFTSV is crucial for prevention and control of SFTS. CRISPR detection can be integrated with isothermal nucleic acid amplification technologies, like enzymatic recombination amplification^5^ and RAA,^6^ to attain highly specific and sensitive detection, showing the potential of developing POCT product.^7^ Here, we developed two CRISPR‐based visual detection methods for SFTSV to fulfil the detection needs in various scenarios.

We screened a conserved sequence of segment L of SFTSV (Table S1) as the target. Ten CRISPR RNAs (crRNAs) were designed from the target sequence and synthesized, as listed in Table S2. The detection efficiency of each crRNA in the CRISPR/Cas12a detection system was individually evaluated (Figure S1). CrRNA2, exhibiting the highest reaction efficiency with a slope ratio of 14.26 between the positive group and negative control, was screened as the optimal one (Table S3).

The RCCF reporter detection system was developed and optimized by integrating CRISPR/Cas12a, RAA and the fluorescent reporter (Figure 1A). The endpoint fluorescence signal was visually detected using a fluorescent excited flashlight. Initially, the entire CRISPR/Cas12a system was validated (Figure 1B,C). The Cas12a nuclease was activated to cleave the reporter in the presence of the positive template, Cas12a and crRNA. To enhance the detection performance, we evaluated various concentrations of Cas12a (Figure 1D) and determined 445 nM is the optimal concentration. Without RAA amplification, the limit of detection (LOD) of the method was 10^9^ copies/reaction (Figure 1E,F), indicating the necessity of introducing a preamplification procedure. We optimized the volume of RAA amplification products added to the CRISPR/Cas12a system and determined that 5 µL was the optimal volume (Figure 1G).

**FIGURE 1:**
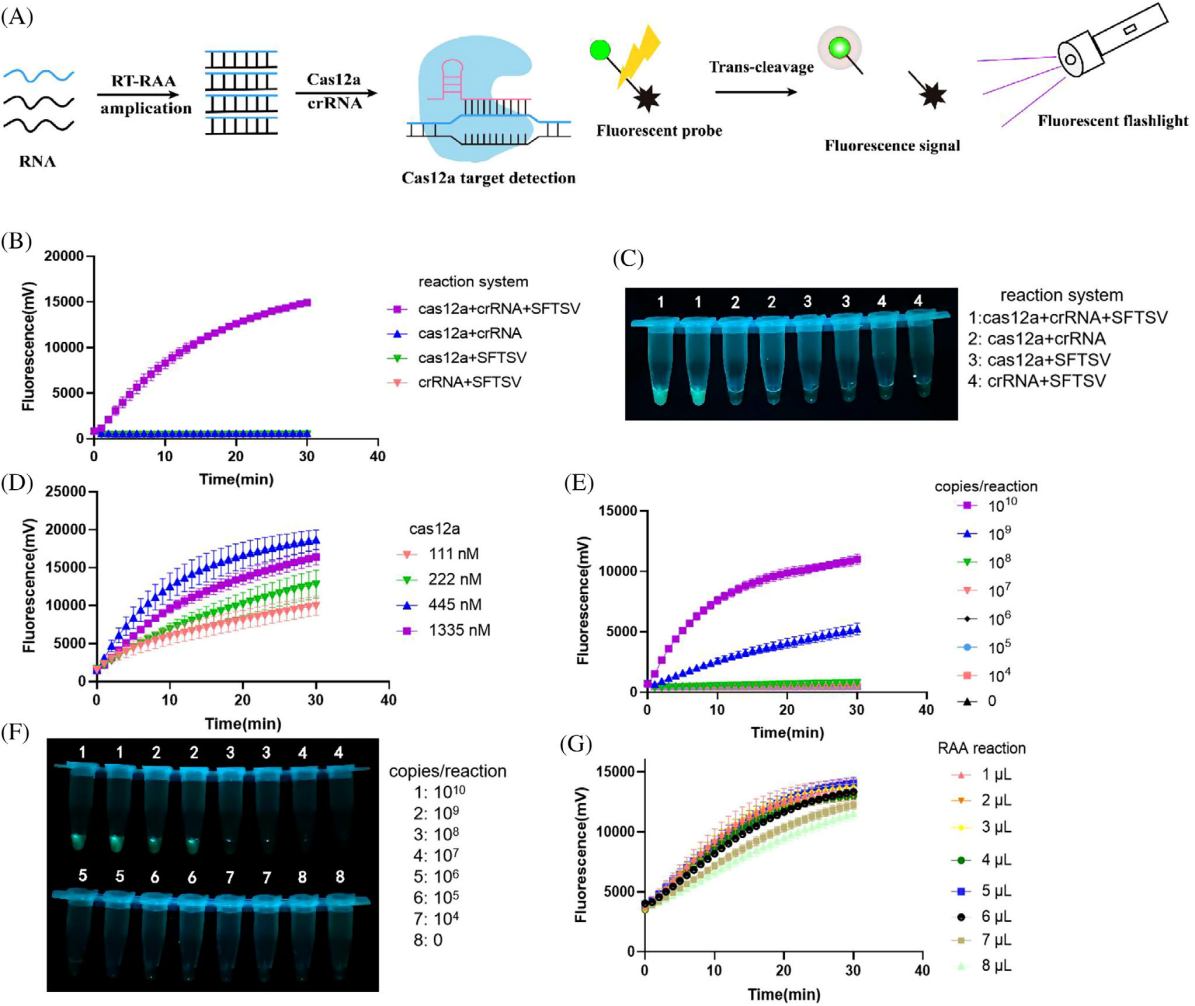
Optimization of the RAA‐CRISPR/Cas12a‐fluorescent (RCCF) visual detection system. (A) Schematic illustration of RCCF **v**isual detection system. The target gene fragment is initially amplified through RT‐recombinase‐aided amplification (RRAA) amplification. In the RCCF system, the amplified target sequence binds to crRNA, activating Cas12a nucleases to *trans*‐cleave ssDNA‐fluorescent reporter. Using a tiny fluorescent flashlight, we can observe the fluorescence signal. (B) Fluorescence signal of the CRISPR/Cas12a detection system under various reaction conditions. (C) Endpoint visualization of the CRISPR/Cas12a system under various reaction conditions. (D) Effects of various concentrations of Cas12a on the CRISPR‐based fluorescence detection. (E and F) Detection of a series of gradient dilutions of severe fever with thrombocytopenia syndrome virus (SFTSV) plasmid using a CRISPR‐based fluorescence detection system without preamplification. (G) Effects of different volumes of RAA amplification products on the performance of the RCCF visual detection system.

Another visual detection system named the RCCD was developed by combining RAA, CRISPR/Cas12a with G‐quadruplex (G4) DNAzyme reporter^8^ (Figures 2A). In the presence of ABTS^2−^ and H_2_O_2_, the peroxidase activity of G4 and hemin can catalyse the oxidation of ABTS^2−^, resulting in a colour change from transparent to dark green, with a maximum absorption wavelength at 405 nm (Figure 2B). The colour‐rendering effect of another substrate, TMB, was also evaluated, but not as well as ABTS (Figure S2). We integrated the G4/hemin DNAzyme into the Cas12a system. The presence of the positive plasmid, Cas12a and crRNA led to the degradation of G4 into fragments (Figure 2C). Consequently, the fragmented G4 failed to interact with hemin to form the G4/hemin DNA enzyme with peroxidase activity, leading to only a slight colour change and low absorption value at 405 nm being observed.

**FIGURE 2:**
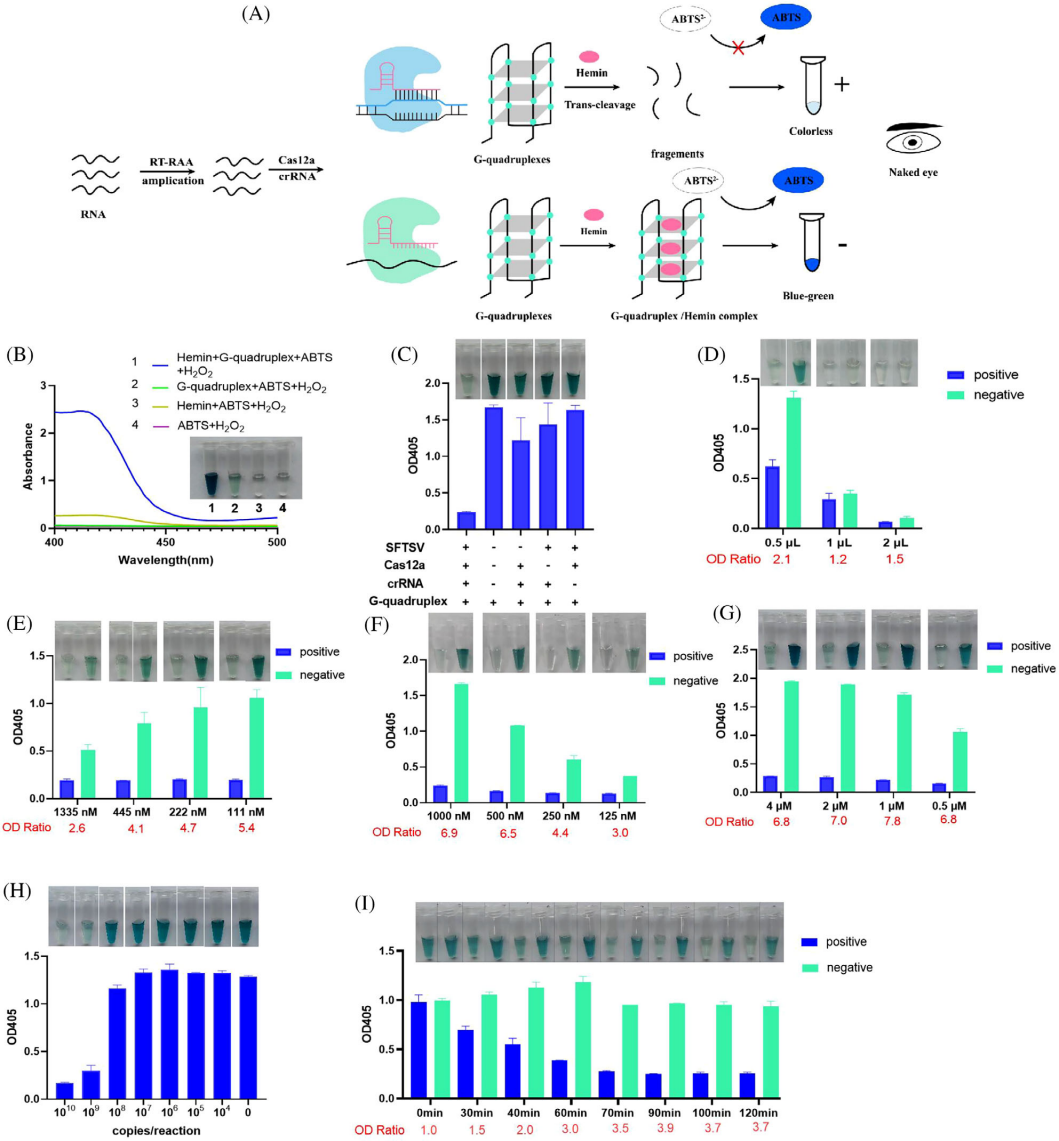
Optimization of the RAA‐CRISPR/Cas12a‐DNAzyme (RCCD) visual detection system. (A) Schematic illustration of RCCD **v**isual detection system. The target gene fragment is initially amplified through RT‐recombinase‐aided amplification (RAA) amplification. The amplified target sequence similarly binds to crRNA, activating the Cas12a nucleases to *trans*‐cleave G‐quadruplex (G4). As a result, the cleaved G4 cannot bind with hemin, which means it loses its peroxidase activity and prevents the catalysis of the ABTS^2–^ colourimetric reaction. Consequently, the solution remains transparent. In the absence of the target gene, G4 is not cleaved and can bind with hemin to form G4/hemin DNA enzyme. This enzyme then facilitates the colour reaction of ABTS^2−^, resulting in a transition from transparency to a blue–green hue. In contrast, when the target gene is present, this colourimetric reaction is inhibited, allowing the colour change to be observed with the naked eye. (B) UV–visible absorption curve of the G4‐catalysed ABTS^2−^‐H_2_O_2_ colour reaction under various reaction systems. (C) UV–visible absorbance values of G4 under various reaction conditions. (D) Effect of different volumes of recombinase‐aided amplification (RAA) amplification products on the RCCD visual detection system. (E) Colour development under various concentrations of Cas12a. (F) Colour development under various concentrations of G4. (G) Colour development under different concentrations of hemin. (H) Detection of a series of gradient dilutions of positive plasmid using CRISPR/Cas12a‐G4 system without preamplification. (I) Evaluation of the efficiency of *trans*‐cleavage G‐quadruplex ssDNA by the Cas12a/crRNA complex over time. OD Ratio = absorption value of negative group at 405 nm/absorption value of positive group at 405 nm.

To improve the detection performance, we optimized the volume of RAA amplification product, Cas12a, and concentrations of G4 as well as the hemin (Figure 2D–G) in the detection system. As a result, 0.5 µL of RAA amplification product, 111 nM of Cas12a, 1000 nM of G4 and 1 µM of hemin were used in the final detection system. The LOD of the detection system without preamplification was determined to be 10^9^ copies/reaction (Figure 2H). We analysed reaction outcomes at various time intervals (Figure 2I). For visual detection, the optimal cleavage reaction time was ascertained to be 70 min.

To assess the LODs of the RCCF and RCCD visualization systems, we used serially diluted SFTSV genomic RNA as templates. The LODs of both RCCF and RCCD systems were 1 copy/reaction (Figure 3A–D). The specificity of RCCF and RCCD visualization systems was evaluated, and both methods exhibit exceptional specificity without recognizing other tick viruses like Alongshan virus or Henan tick virus (Figure 3E,F).

**FIGURE 3:**
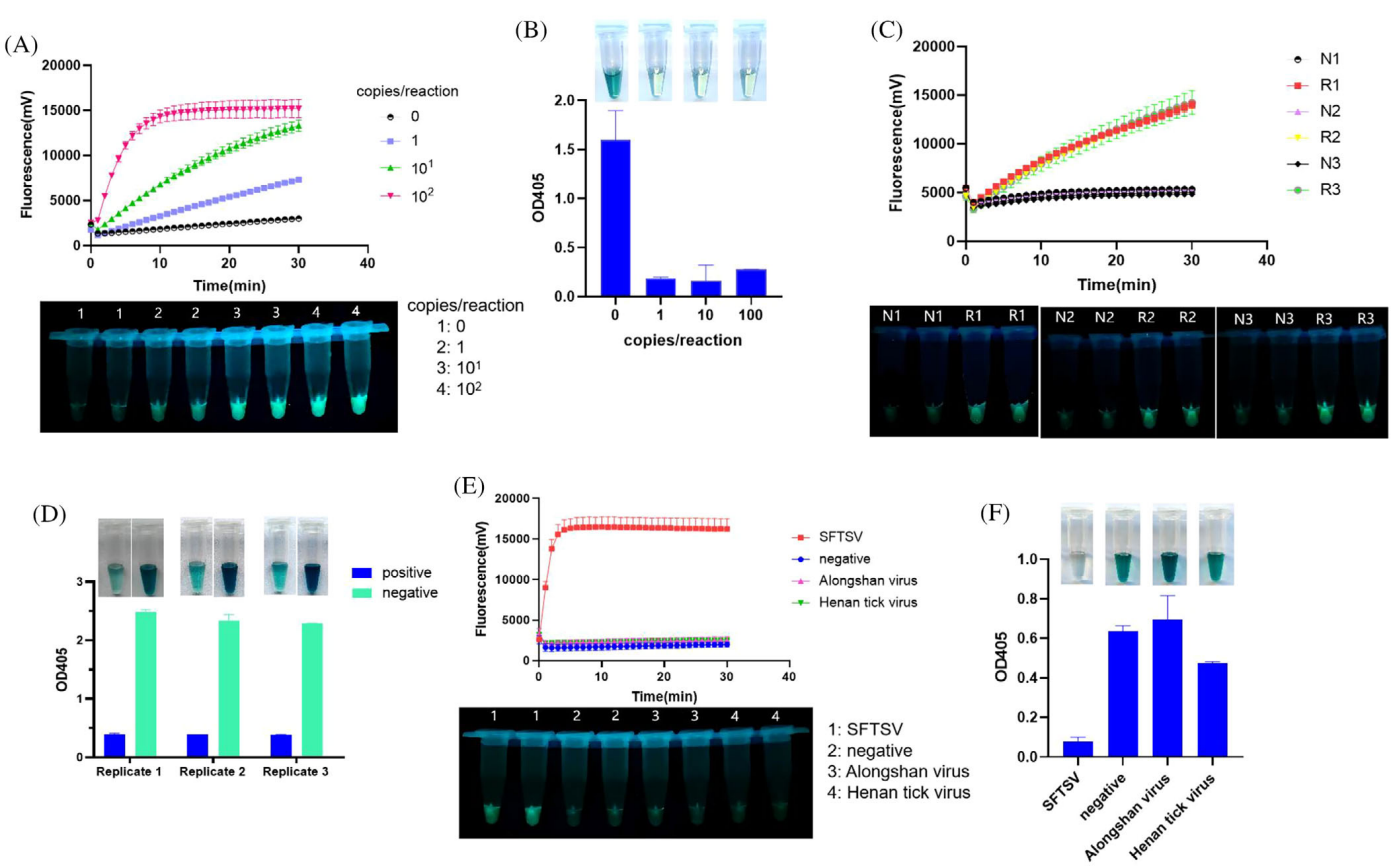
Limit of detection and specificity analysis of the RAA‐CRISPR/Cas12a‐fluorescent (RCCF) and RAA‐CRISPR/Cas12a‐DNAzyme (RCCD) visualization systems. (A) Limit of detection of the RCCF visualization system in detecting severe fever with thrombocytopenia syndrome virus (SFTSV) genomic RNA. (B) Limit of detection of the RCCD visualization system in detecting SFTSV genomic RNA. (C) Reproducibility assessment of the RCCF visualization system in the detection of SFTSV genomic RNA. R1, R2 and R3 indicate three replicates of RNA samples of 1 copy/reaction. N1, N2 and N3 indicate three replicates of negative samples. (D) Reproducibility assessment of the RCCD visualization system in the detection of SFTSV genomic RNA. Replicate 1, Replicate 2 and Replicate 3 indicate three replicates. Positive samples were 1 copy/reaction RNA samples. (E) Specificity of the RCCF visualization system in detecting genomic RNA of SFTSV and other tick‐borne viruses. (F) Specificity of the RCCD visualization system in detecting genomic RNA of SFTSV genomic RNA and other tick‐borne viruses.

To evaluate the actual performances of the developed systems, RNAs in 66 clinical serum samples (33 positive samples and 33 negative samples) and 4 tick homogenates (2 positive samples and 2 negative samples) were extracted and used as templates (all the 70 samples were tested by RT‐PCR, Table S4). These serum samples, which tested negative clinically, were obtained from patients who had previously tested positive for tick bites. Metagenomic sequencing of these samples identified the presence of multiple viruses, including human pegivirus, Okutama tick virus, Dabieshan tick virus and Jingmen tick virus. The results demonstrated that both the RCCF and RCCD visualization systems can effectively analyse SFTSV in clinical samples and tick homogenates, with a sensitivity reaching 100% and a specificity attaining 100% (Figure 4A–C).

**FIGURE 4:**
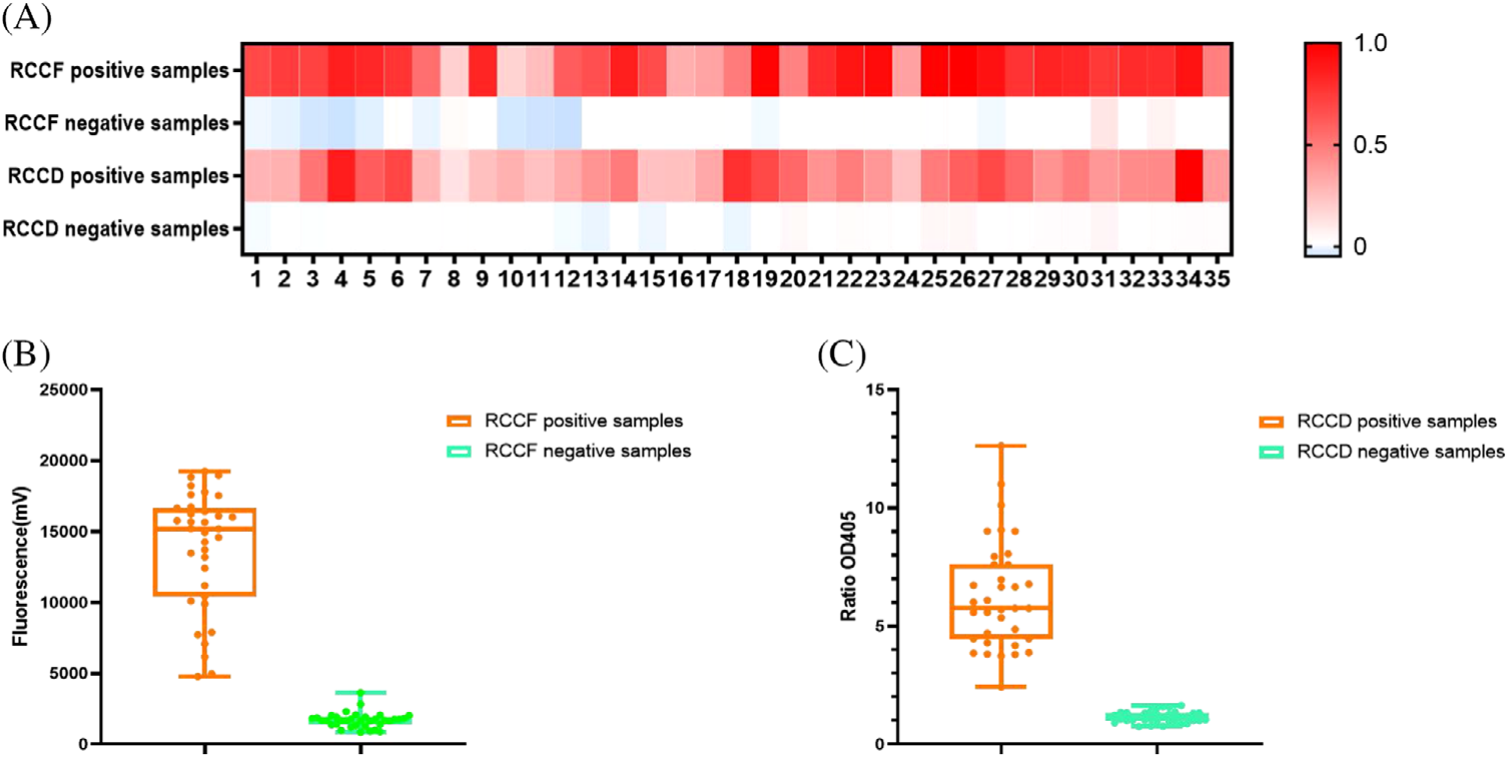
Performances of the detection systems in detecting clinical sera and tick samples. (A) Heatmap of 70 samples detected by RAA‐CRISPR/Cas12a‐fluorescent (RCCF) and RAA‐CRISPR/Cas12a‐DNAzyme (RCCD), respectively. Under RCCF system, the fluorescent signal of each sample was normalized against the negative control of the same batch. Under RCCD system, the OD405 ratio of negative control and positive samples were normalized. Samples 1–33 were clinical serums. Samples 34 and 35 were tick homogenates. (B) Box and whisker of 70 real samples detected by RCCF. (C) Box and whisker of 70 real samples detected by RCCF. Ratio OD405 = absorption value of the negative group at 405 nm/absorption value of the sample group at 405 nm.

The detection performances of the established RCCD and RCCF are shown in Table S5, compared with previously reported CRISPR‐based detection methods.^9^ We have greatly improved the performance of SFTSV detection in many aspects such as LOD, cost‐effectiveness and batch testing. However, both methods have limitations. They still require two separate processes: a preamplification procedure and the CRISPR development procedure, which can be time‐consuming. Further optimization is necessary.

In summary, we successfully established two sensitive and visual detection methods for SFTSV by combining RAA and CRISPR detection. The RCCF method, based on a portable excitation light source, is free from the limitations of conventional fluorescent detectors for rapid detection of SFTSV. The RCCD method requires neither an excitation light source to achieve visual detection by the naked eye nor expensive fluorescence‐modified reporter groups, which is suitable for laboratory or field detection in resource‐limited settings. Moreover, they are suitable for batch detection, overcoming the shortcomings of traditional visual detection using lateral chromatography, which cannot be detected in batches. These methods meet detection requirements across different scenarios for SFTS and have broad application prospects.

## AUTHOR CONTRIBUTIONS

The study was conceived and the experimental protocol was designed by Junhu Wang and Yong Qi. Junhu Wang, Ming Yue, Qiong Chen, Yixin Ge, Haiming Yi, Hongming Wang and Sunjie Yang conducted the experiments. Junhu Wang, Yuexi Li and Jin Zhu analysed the data. The paper was written by Junhu Wang, Ming Yue, Yuexi Li and Jin Zhu. All authors were involved in the revision of the article and granted final approval for the version to be published.

## CONFLICT OF INTEREST STATEMENT

The authors declare no conflicts of interest.

## ETHICS STATEMENT

The Ethics Committee of Huadong Research Institute for Medicine and Biotechniques approved the utilization of clinical samples.

## CONSENT

We obtained written informed consent from the patients and their information was de‐identified.

## Supporting information



Supporting Information

## Data Availability

Upon reasonable request, the datasets were available from the corresponding author.
