# Supplementary material for: Field‐deployable, ultrasensitive and visual detection systems targeting severe fever with thrombocytopenia syndrome virus (Dabie bandavirus) based on CRISPR/Cas12a and DNAzyme
Source: Clin Transl Med. 2025 Jul 3;15(7):e70393. doi: 10.1002/ctm2.70393 (PMC12226718; doi:10.1002/ctm2.70393)
Supplement: Supplementary file 1 — Supporting Information [file CTM2-15-e70393-s001.docx]

**Supplementary materials**

**MATERIALS AND METHODS**

**1 Sample nucleic acid extraction**

Genomic RNAs of SFTSV JS14*,* Alongshan virus isolate Liaoning, Henan tick virus isolate CLCM-130, patient serum samples and tick homogenates were extracted using a Viral RNA/DNA Extraction Kit (TaKaRa, Beijing, China). The concentrations of the extracted RNAs were measured by a microspectrophotometer (Beijing KAIAO, Beijing, China). All the genomic RNAs (SFTSV JS14*,* Alongshan virus isolate Liaoning, and Henan tick virus isolate CLCM-130) were diluted with elution buffer to 10 ng/μL.

**2 Specific gene sequence screening**

To screen specific RNA fragments of SFTSV, the genome sequences of all species within the genus *Bandaviru*s were collected and aligned in the Mauve software (The Darling lab at the University of Technology Sydney). All the specific sequences of over 300 bp in SFTSV were selected and further aligned with all the public genome sequences of SFTSV strains using the BLAST software online (http://blast.ncbi.nlm.nih.gov/), to ensure the sequence is conserved within the species. To test the out-of-genus specificity of the selected sequences, they were aligned with all the gene sequences from non-SFTSV species in GenBank. The sequence showing the highest specificity and conservation was selected as the target sequence, chemically synthesized by Sangon Biotech Company (Shanghai, China), and linked to pUC57 plasmid to construct a positive template for further detection method development.

**3 crRNA screening and optimization**

A series of crRNA sequences were designed according to the restricted PAM sequences for Cas12a and chemically synthesized by Sangon (Shanghai, China). The fluorescent CRISPR reaction system included 1 µL LbCas12a (1 µM, GenScript, Nanjing, China), 1 µL crRNA (1 µM), 1 µL FAM-TTTTTTTTTTTT-BHQ1 reporter(1 µM), 14 µL DEPC water, 2 µL NEB buffer 2.1 (NEB, Beijing, China), positive plasmids (10^10^ copies) and incubated at 37°C. Real-time fluorescence signals were measured by a F1620 fluorescent reader (Qitian Gene, Wuxi, China) at 20-second intervals for 30 min. A negative control using plasmid pUC57 as the template was conducted in each test. The dynamic change of fluorescence value with reaction time was plotted and the slope was calculated. A higher slope indicated a higher detection efficiency. The optimal crRNA was selected with the highest slope ratio between the positive group and the negative group.

**4 Reverse transcription-RAA assay**

The SFTSV RAA was generated using a reverse transcription-RAA (RT-RAA) nucleic acid amplification kit (Qitian Gene) according to the manufacturer’s instructions. Briefly, the 50 μL reaction volume contained 25 μL of Buffer V, 2 μL (10 μM) of each upstream primer, downstream primer, 1 μL template (Table 1), 15 μL ddH2O and 5 μL magnesium acetate. The mixture was shaken and incubated at 37℃ for 30 min.

**5 CRISPR-G4 colorimetric reaction development**

In the initial CRISPR-based detection system, G4 ssDNA was used to replace the fluorescent reporter. After incubation, hemin (Sangon, Shanghai, China) and KCl were added to activate the peroxidase activity. Then, an appropriate amount of color-developing substrate was added, and the mixture was incubated for another 5 min at 37°C. The color change of the solution was observed by naked eyes. In addition, the absorbance of the solution was measured by a [Multiskan](https://www.thermofisher.cn/order/catalog/product/cn/en/1410101) FC microplate photometer (Thermo, Waltham, America). Various color-developing substrates were evaluated for their suitable use in the system. We conducted a comparative analysis of the color development between TMB and ABTS. As shown in Figure S3, the color difference between positive and negative groups was more pronounced with ABTS as the substrate. Also, concentrations of various components in the reaction system were optimized.

**6 RCCF detection assay development**

The fluorescent CRISPR reaction was combined with the RT-RAA assay to construct a detection method. Briefly, the target fragment was amplified using the RT-RAA assay. Then, various volumes of the amplification product were added to the optimized fluorescent CRISPR reaction system for detection by the F1620 fluorescent reader. Visual detection was accomplished by illuminating the tubes through LUYOR-3280 Fluorescent flashlight (Luyang, Shanghai, China).

**7 RCCD** **detection assay development**

The RT-RAA assay was combined with the CRISPR-G4 reaction to construct a visual detection method. Briefly, the target fragment was amplified using the RT-RAA assay. Here, a basic RT-RAA Nucleic Acid Amplification Kit (Qitian Gene) was used without the probe added. Then, various volumes of the amplification product were added to the optimized CRISPR-G4 colorimetric reaction system for colorimetric detection.

**8 Limit of detection and specificity evaluation**

The limits of detection (LODs) of the assays were measured using serially diluted genomic RNAs (with concentrations of 10^2^, 10, and 1 copies/µL) of SFTSV in elution buffer as templates, and the minimum concentration that could be detected was the LOD. The detection of the minimum concentration was confirmed by three additional assays. Genomic RNAs of Alongshan virus and Henantick virus were used as templates in the constructed detection assays for specificity evaluation. Elution buffer served as the template of the negative control in both experiments. Each reaction was performed with two replicates.

**9 Performance of the assays in detecting clinical and tick samples**

Samples included 66 clinical serum samples and 4 tick homogenates. Out of the 66 clinical serum samples, 33 samples were collected from patients diagnosed with SFTS based on both clinical symptoms and RT-PCR test, while the remaining 33 samples were obtained from tick-bite febrile patients who tested nucleic acid-negative for SFTSV. Out of the 4 tick homogenates, 2 were SFTSV nucleic acid-positive and the other 2 were SFTSV nucleic acid-negative. For RT-PCR test, a commercial kit (Daan Gene Co., Ltd, China) was used. Genomic RNAs of the patient serum samples and tick homogenates were used as templates in the constructed assays for test their performances.

**10 Data analysis**

All data generated in the experiment were processed by GraphPad Prism 8.3.0 to generate curve and bar graphs.

**TABLE S1 Blast alignment results of the selected sequence from segment L (accession No. NC_043450.1, from base 5641 to 6240) with the corresponding sequences in other SFTSV isolates.**

| Accession | Query Cover | Per. ident | Acc. Len |
| --- | --- | --- | --- |
| PP239063.1 | 100% | 100 | 6255 |
| NC_043450.1 | 100% | 100 | 6255 |
| OM453335.1 | 100% | 99.83 | 6368 |
| OM453402.1 | 100% | 99.83 | 6368 |
| KC292329.1 | 100% | 99.83 | 6368 |
| OM453027.1 | 100% | 99.83 | 6368 |
| OM453093.1 | 100% | 99.83 | 6368 |
| HQ419227.1 | 100% | 99.83 | 6368 |
| OM453544.1 | 100% | 99.83 | 6368 |
| OM453171.1 | 100% | 99.83 | 6368 |
| OM453255.1 | 100% | 99.83 | 6368 |
| OM453571.1 | 100% | 99.83 | 6368 |
| OM452983.1 | 100% | 99.83 | 6368 |
| OP652094.1 | 100% | 99.83 | 6348 |
| OM453075.1 | 100% | 99.83 | 6368 |
| OM453083.1 | 100% | 99.83 | 6368 |
| OM453184.1 | 100% | 99.83 | 6368 |
| OM453020.1 | 100% | 99.83 | 6368 |
| OM453186.1 | 100% | 99.83 | 6368 |
| OM453470.1 | 100% | 99.83 | 6368 |
| OM453041.1 | 100% | 99.83 | 6368 |
| OM453418.1 | 100% | 99.83 | 6368 |
| OM452980.1 | 100% | 99.83 | 6368 |
| KF711862.1 | 100% | 99.83 | 6368 |
| KF356546.1 | 100% | 99.83 | 6344 |
| OM453243.1 | 100% | 99.83 | 6368 |
| OM453114.1 | 100% | 99.83 | 6368 |
| OM453167.1 | 100% | 99.66 | 6368 |
| OM453607.1 | 100% | 99.66 | 6368 |
| OM453238.1 | 100% | 99.66 | 6368 |
| OM453286.1 | 100% | 99.66 | 6368 |
| MK513924.1 | 100% | 99.66 | 6368 |
| KC292348.1 | 100% | 99.66 | 6368 |
| KF791958.1 | 100% | 99.66 | 6368 |
| OM452943.1 | 100% | 99.66 | 6368 |
| OM453047.1 | 100% | 99.66 | 6368 |
| OM453058.1 | 100% | 99.66 | 6368 |
| MT114240.1 | 100% | 99.66 | 6368 |
| OM453477.1 | 100% | 99.66 | 6368 |
| OM453231.1 | 100% | 99.66 | 6368 |
| OM452960.1 | 100% | 99.66 | 6368 |
| OM452961.1 | 100% | 99.66 | 6368 |
| KC292328.1 | 100% | 99.66 | 6368 |
| OM453333.1 | 100% | 99.66 | 6368 |
| JQ341188.1 | 100% | 99.66 | 6368 |
| OM453347.1 | 100% | 99.66 | 6368 |
| OM453085.1 | 100% | 99.66 | 6368 |
| OM453557.1 | 100% | 99.66 | 6368 |
| OM452974.1 | 100% | 99.66 | 6368 |
| OM453279.1 | 100% | 99.66 | 6368 |
| OM453016.1 | 100% | 99.66 | 6368 |
| OM453176.1 | 100% | 99.66 | 6368 |
| OM453236.1 | 100% | 99.66 | 6368 |
| OM453513.1 | 100% | 99.66 | 6368 |
| OM453245.1 | 100% | 99.66 | 6368 |
| OM453503.1 | 100% | 99.66 | 6368 |
| OM453419.1 | 100% | 99.66 | 6368 |
| OM452998.1 | 100% | 99.66 | 6368 |
| OM453056.1 | 100% | 99.66 | 6368 |
| OM453480.1 | 100% | 99.66 | 6368 |
| OM453265.1 | 100% | 99.66 | 6368 |
| MN509899.2 | 100% | 99.66 | 6368 |
| OM452999.1 | 100% | 99.66 | 6368 |
| OM453553.1 | 100% | 99.66 | 6368 |
| OM453097.1 | 100% | 99.66 | 6368 |
| MN509848.2 | 100% | 99.66 | 6368 |
| OM453462.1 | 100% | 99.66 | 6368 |
| OM452944.1 | 100% | 99.66 | 6368 |
| OM453121.1 | 100% | 99.66 | 6368 |
| OM453270.1 | 100% | 99.66 | 6368 |
| OM453048.1 | 100% | 99.66 | 6368 |
| OM452955.1 | 100% | 99.66 | 6368 |
| OM453215.1 | 100% | 99.66 | 6368 |
| OM453595.1 | 100% | 99.66 | 6368 |
| OM453065.1 | 100% | 99.66 | 6368 |
| OM453360.1 | 100% | 99.66 | 6368 |
| OM452953.1 | 100% | 99.66 | 6368 |
| OM453570.1 | 100% | 99.66 | 6368 |
| OM453312.1 | 100% | 99.66 | 6368 |
| OM453372.1 | 100% | 99.66 | 6368 |
| OM453447.1 | 100% | 99.66 | 6368 |
| MN509903.2 | 100% | 99.66 | 6368 |
| OM453468.1 | 100% | 99.66 | 6368 |
| OM453257.1 | 100% | 99.66 | 6368 |
| OM453437.1 | 100% | 99.66 | 6368 |
| OM453449.1 | 100% | 99.66 | 6368 |
| OM453049.1 | 100% | 99.66 | 6368 |
| OM453239.1 | 100% | 99.66 | 6368 |
| OM453013.1 | 100% | 99.66 | 6368 |
| OM453371.1 | 100% | 99.66 | 6368 |
| OM453133.1 | 100% | 99.66 | 6368 |
| OM453308.1 | 100% | 99.66 | 6368 |
| MN509950.2 | 100% | 99.66 | 6368 |
| OM453120.1 | 100% | 99.66 | 6368 |
| KF711861.1 | 100% | 99.66 | 6368 |
| OM453033.1 | 100% | 99.66 | 6368 |
| OM453354.1 | 100% | 99.66 | 6368 |
| MN509991.2 | 100% | 99.66 | 6368 |
| OM453399.1 | 100% | 99.66 | 6368 |
| OM453095.1 | 100% | 99.66 | 6368 |
| OM453262.1 | 100% | 99.66 | 6368 |
| OM453260.1 | 100% | 99.66 | 6368 |
| OM453340.1 | 100% | 99.66 | 6368 |
| OM453298.1 | 100% | 99.66 | 6368 |
| OM453542.1 | 100% | 99.48 | 6368 |
| OM453162.1 | 100% | 99.48 | 6368 |
| OM453507.1 | 100% | 99.48 | 6368 |
| OM453276.1 | 100% | 99.48 | 6368 |
| OM453481.1 | 100% | 99.48 | 6368 |
| OM453071.1 | 100% | 99.48 | 6368 |
| OM453458.1 | 100% | 99.48 | 6368 |
| OM453508.1 | 100% | 99.48 | 6368 |
| OM453204.1 | 100% | 99.48 | 6368 |
| OM453259.1 | 100% | 99.48 | 6368 |
| OM453105.1 | 100% | 99.48 | 6368 |
| OM453258.1 | 100% | 99.48 | 6368 |
| OM453109.1 | 100% | 99.48 | 6368 |
| OM453247.1 | 100% | 99.48 | 6368 |
| OM453043.1 | 100% | 99.48 | 6368 |
| OM453491.1 | 100% | 99.48 | 6368 |
| OM453115.1 | 100% | 99.48 | 6368 |
| OM453209.1 | 100% | 99.48 | 6368 |
| OM453501.1 | 100% | 99.48 | 6368 |
| OM453471.1 | 100% | 99.48 | 6368 |
| OM453101.1 | 100% | 99.48 | 6368 |
| OM452952.1 | 100% | 99.48 | 6368 |
| OM453089.1 | 100% | 99.48 | 6368 |
| OM453002.1 | 100% | 99.48 | 6368 |
| OP652089.1 | 100% | 99.48 | 6342 |
| OM453229.1 | 100% | 99.48 | 6368 |
| OM453400.1 | 100% | 99.48 | 6368 |
| OM453369.1 | 100% | 99.48 | 6368 |
| OM453568.1 | 100% | 99.48 | 6368 |
| OM453346.1 | 100% | 99.48 | 6368 |
| OM453198.1 | 100% | 99.48 | 6368 |
| KC292349.1 | 100% | 99.48 | 6368 |
| OP652112.1 | 100% | 99.48 | 6340 |
| OM452990.1 | 100% | 99.48 | 6368 |
| OM453062.1 | 100% | 99.48 | 6368 |
| OM453550.1 | 100% | 99.48 | 6368 |
| OM453080.1 | 100% | 99.48 | 6368 |
| OM453496.1 | 100% | 99.48 | 6368 |
| HQ141598.1 | 100% | 99.48 | 6368 |
| OM453536.1 | 100% | 99.48 | 6368 |
| KY965118.1 | 100% | 99.48 | 6368 |
| MT114239.1 | 100% | 99.48 | 6368 |
| OM453142.1 | 100% | 99.48 | 6368 |
| OM453494.1 | 100% | 99.48 | 6368 |
| OM453398.1 | 100% | 99.48 | 6368 |
| MF045954.1 | 100% | 99.48 | 6368 |
| OM453432.1 | 100% | 99.48 | 6368 |
| MN509929.2 | 100% | 99.48 | 6368 |
| OM453295.1 | 100% | 99.48 | 6368 |
| MF045950.1 | 100% | 99.48 | 6368 |
| OM453180.1 | 100% | 99.48 | 6368 |
| OM453436.1 | 100% | 99.48 | 6368 |
| OM453448.1 | 100% | 99.48 | 6368 |
| MK355635.1 | 100% | 99.48 | 6368 |
| OM452969.1 | 100% | 99.48 | 6368 |
| OM453290.1 | 100% | 99.48 | 6368 |
| OM453311.1 | 100% | 99.48 | 6368 |
| OM453025.1 | 100% | 99.48 | 6368 |
| OM453074.1 | 100% | 99.48 | 6368 |
| MT320799.1 | 99% | 99.48 | 6368 |
| OM453092.1 | 100% | 99.31 | 6368 |
| HQ171190.1 | 100% | 99.31 | 6368 |
| OQ388746.1 | 100% | 99.31 | 6368 |
| OM453283.1 | 100% | 99.31 | 6368 |
| OM453151.1 | 100% | 99.31 | 6368 |
| OM453034.1 | 100% | 99.31 | 6368 |
| OM453475.1 | 100% | 99.31 | 6368 |
| OM453217.1 | 100% | 99.31 | 6368 |
| OM453272.1 | 100% | 99.31 | 6368 |
| OM453362.1 | 100% | 99.31 | 6368 |
| OM453022.1 | 100% | 99.31 | 6368 |
| OM453060.1 | 100% | 99.31 | 6368 |
| OM453254.1 | 100% | 99.31 | 6368 |
| OM453112.1 | 100% | 99.31 | 6368 |
| OQ413509.1 | 100% | 99.31 | 6355 |
| OQ388754.1 | 100% | 99.31 | 6368 |
| KY933699.1 | 100% | 99.31 | 6368 |
| OM453129.1 | 100% | 99.31 | 6368 |
| OM453411.1 | 100% | 99.31 | 6368 |
| MT005223.1 | 100% | 99.31 | 6368 |
| OM452947.1 | 100% | 99.31 | 6368 |
| OM453252.1 | 100% | 99.31 | 6368 |
| MT114236.1 | 100% | 99.14 | 6368 |
| MT320796.1 | 100% | 99.14 | 6368 |
| OM453009.1 | 100% | 99.14 | 6368 |
| MK513927.1 | 100% | 99.14 | 6366 |
| OM453366.1 | 100% | 99.14 | 6368 |
| OM453170.1 | 100% | 99.14 | 6368 |
| OM453057.1 | 100% | 99.14 | 6368 |
| KF711863.1 | 100% | 98.97 | 6368 |
| OM453014.1 | 100% | 98.97 | 6368 |
| OQ938864.1 | 100% | 98.79 | 6299 |
| OQ938868.1 | 100% | 98.79 | 6368 |
| PP975924.1 | 100% | 98.79 | 6251 |
| MZ773015.1 | 100% | 98.79 | 6368 |
| AB985644.1 | 100% | 98.79 | 1771 |
| MZ773027.1 | 100% | 98.79 | 6368 |
| OM453401.1 | 100% | 98.79 | 6368 |
| MT005202.1 | 100% | 98.79 | 6368 |
| PP975923.1 | 100% | 98.79 | 6255 |
| PP706855.1 | 100% | 98.79 | 6299 |
| OQ938867.1 | 100% | 98.79 | 6368 |
| MG736981.1 | 100% | 98.79 | 6255 |
| PP975925.1 | 100% | 98.79 | 6255 |
| PP706857.1 | 100% | 98.79 | 6299 |
| MZ773021.1 | 100% | 98.79 | 6368 |
| PP706856.1 | 100% | 98.79 | 6299 |
| OQ938866.1 | 100% | 98.79 | 6368 |
| MZ773025.1 | 100% | 98.79 | 6368 |
| PP706887.1 | 100% | 98.62 | 6299 |
| HQ830169.1 | 100% | 98.62 | 6368 |
| KC505141.1 | 100% | 98.62 | 6368 |
| PP706829.1 | 100% | 98.62 | 6299 |
| KR230757.1 | 100% | 98.62 | 6368 |
| HQ830163.1 | 100% | 98.62 | 6368 |
| OQ938863.1 | 100% | 98.62 | 6308 |
| JF837593.1 | 100% | 98.62 | 6368 |
| OQ938865.1 | 100% | 98.62 | 6306 |
| KY362305.1 | 100% | 98.62 | 6368 |
| KY362297.1 | 100% | 98.62 | 6368 |
| KC505144.1 | 100% | 98.62 | 6368 |
| PP488495.1 | 100% | 98.62 | 6368 |
| KC505123.1 | 100% | 98.62 | 6368 |
| OR416547.1 | 100% | 98.62 | 6346 |
| KY362294.1 | 100% | 98.62 | 6368 |
| KC505138.1 | 100% | 98.62 | 6368 |
| PP706845.1 | 100% | 98.62 | 6299 |
| PP706852.1 | 100% | 98.62 | 6299 |
| LC570786.1 | 100% | 98.62 | 6324 |
| KY362299.1 | 100% | 98.62 | 6368 |
| HQ141592.1 | 100% | 98.62 | 6368 |
| PP706878.1 | 100% | 98.45 | 6299 |
| MZ773023.1 | 100% | 98.45 | 6368 |
| KY362316.1 | 100% | 98.45 | 6368 |
| KY273267.1 | 100% | 98.45 | 6368 |
| PP706884.1 | 100% | 98.45 | 6299 |
| MT005217.1 | 100% | 98.45 | 6368 |
| PP706888.1 | 100% | 98.45 | 6299 |
| MK355643.1 | 100% | 98.45 | 6368 |
| PP706854.1 | 100% | 98.45 | 6299 |
| PP706828.1 | 100% | 98.45 | 6299 |
| PP706873.1 | 100% | 98.45 | 6299 |
| OR416562.1 | 100% | 98.45 | 6364 |
| MT005220.1 | 100% | 98.45 | 6368 |
| PP706837.1 | 100% | 98.45 | 6299 |
| MZ773026.1 | 100% | 98.45 | 6368 |

**TABLE S2 Sequences of the primers, crRNAs, and reporters employed in this study**

| Names | Sequences (5’-3’) |
| --- | --- |
| RAA-F | TCAGTTCTCCTGGCAAATGATAGGAAGACCCAAGG |
| RAA-R | CTCCAAACTCTTCCACCTCAGCAGACCACAA |
| crRNA1 | UAAUUUCUACUAAGUGUAGAUCTCTWAGAAAGCAAGGGYTGATGAGGTCAA |
| crRNA2 | UAAUUUCUACUAAGUGUAGAUTCAGTTCTCCTGGCAAATGATAGGAAGACCCAAGG |
| crRNA3 | UAAUUUCUACUAAGUGUAGAUCAGGGACTGTCTCGAGGGTTCTCTAAGAAAGCAAG |
| crRNA4 | UAAUUUCUACUAAGUGUAGAUTCGAGGGTTCTCTAAGAAAGCAAGGGCTGATGAG |
| crRNA5 | UAAUUUCUACUAAGUGUAGAUCAGACCACAAGATAGGTGCRCCATCAAAGA |
| crRNA6 | UAAUUUCUACUAAGUGUAGAUCTCACAGCCACCACTCCTTCTCCAAACTCT |
| crRNA7 | UAAUUUCUACUAAGUGUAGAUCTCCAAACTCTTCCACCTCAGCAGACCACAA |
| crRNA8 | UAAUUUCUACUAAGUGUAGAUACCACTCCTTCTCCAAACTCTTCCACCTCA |
| crRNA9 | UAAUUUCUACUAAGUGUAGAUCTCTWAGAAAGCAAGGGYTGATGAGGTCAA |
| crRNA10 | UAAUUUCUACUAAGUGUAGAUTCAGTTCTCCTGGCAAATGATAGGAAGACCCAAGG |
| FAM-12T-BHQ1 reporter | 5`6-FAM -TTTTTTTTTTTT-BHQ1-3’ |
| G-rich ssDNA-4 | TGGGTAGGGCGGGTTGGGAAA |

The complementary sequences are indicated by underline.

**TABLE S3 Evaluation of reaction efficiencies of the 10 crRNAs in the CRISPR-based detection system.**

| Names | Slope (positive group) | Slope (negative group) | Slope ratio (positive/ negative) |
| --- | --- | --- | --- |
| crRNA1 | 281.2 | 29.68 | 9.47 |
| crRNA2 | 317.1 | 22.24 | 14.26 |
| crRNA3 | 138.3 | 34.84 | 3.97 |
| crRNA4 | 116.6 | 21.42 | 5.44 |
| crRNA5 | 121.3 | 15.43 | 7.86 |
| crRNA6 | 53.68 | 23.89 | 2.25 |
| crRNA7 | 236.2 | 34.29 | 6.89 |
| crRNA8 | 93.46 | 18.36 | 5.09 |
| crRNA9 | 106.4 | 25.17 | 4.23 |
| crRNA10 | 94.76 | 14.41 | 6.58 |

The reaction efficiency is determined by the ratio of the slopes of two linear fitting curves.

**TABLE S4 RT-PCR results for 66 clinical serum samples and 4 tick homogenates**

| Names | CT value | SFTSV RNA (copies/µL)) |
| --- | --- | --- |
| P-1 | 35.2 | 3.2 |
| P-2 | 34.0 | 7.1 |
| P-3 | 34.9 | 4.1 |
| P-4 | 34.7 | 4.6 |
| P-5 | 34.9 | 4.0 |
| P-6 | 35.3 | 2.9 |
| P-7 | 26.1 | 1230.0 |
| P-8 | 20.4 | 100000.0 |
| P-9 | 23.7 | 10400.0 |
| P-10 | 26.5 | 2030.0 |
| P-11 | 26.8 | 1530.0 |
| P-12 | 23.9 | 3760.0 |
| P-13 | 24.6 | 2080.0 |
| P-14 | 21.0 | 27100.0 |
| P-15 | 21.6 | 16900.0 |
| P-16 | 28.6 | 179.0 |
| P-17 | 28.3 | 223.0 |
| P-18 | 21.6 | 17800.0 |
| P-19 | 11.1 | 29100000.0 |
| P-20 | 21.9 | 14600.0 |
| P-21 | 25.0 | 1870.0 |
| P-22 | 21.5 | 18100.0 |
| P-23 | 17.0 | 353000.0 |
| P-24 | 23.1 | 6590.0 |
| P-25 | 25.7 | 1380.0 |
| P-26 | 22.2 | 25200.0 |
| P-27 | 29.1 | 79.2 |
| P-28 | 23.8 | 6980.0 |
| P-29 | 19.0 | 380000.0 |
| P-30 | 31.8 | 11.6 |
| P-31 | 28.8 | 112.0 |
| P-32 | 25.4 | 2190.0 |
| P-33 | 30.0 | 45.4 |
| P-34 | 35.6 | 1.5 |
| P-35 | 33.2 | 7.6 |
| N-1 | N.D | N.D |
| N-2 | N.D | N.D |
| N-3 | N.D | N.D |
| N-4 | N.D | N.D |
| N-5 | N.D | N.D |
| N-6 | N.D | N.D |
| N-7 | N.D | N.D |
| N-8 | N.D | N.D |
| N-9 | N.D | N.D |
| N-10 | N.D | N.D |
| N-11 | N.D | N.D |
| N-12 | N.D | N.D |
| N-13 | N.D | N.D |
| N-14 | N.D | N.D |
| N-15 | N.D | N.D |
| N-16 | N.D | N.D |
| N-17 | N.D | N.D |
| N-18 | N.D | N.D |
| N-19 | N.D | N.D |
| N-20 | N.D | N.D |
| N-21 | N.D | N.D |
| N-22 | N.D | N.D |
| N-23 | N.D | N.D |
| N-24 | N.D | N.D |
| N-25 | N.D | N.D |
| N-26 | N.D | N.D |
| N-27 | N.D | N.D |
| N-28 | N.D | N.D |
| N-29 | N.D | N.D |
| N-30 | N.D | N.D |
| N-31 | N.D | N.D |
| N-32 | N.D | N.D |
| N-33 | N.D | N.D |
| N-34 | N.D | N.D |
| N-35 | N.D | N.D |

N.D stands for "Not Detected". Samples from P1 to P33 were clinical serum samples. P34 and P35 were tick homogenates. Samples from N1 to N33 were clinical serum samples. N34 and N35 were tick homogenates.

**TABLE S5. Comparison of the established RCCD and RCCF with previously reported CRISPR-based detection methods**

| Parameters | RCCF | RCCD | CRISPR-lateral flow test | CRISPR-fluorescent reporter |
| --- | --- | --- | --- | --- |
| Reaction time | 50-60 min | 90-100 min | 52 min | 50 min |
| Limit of detection | 1 copies/reaction | 1 copies/reaction | 100 copies/reaction | 100 copies/reaction |
| Specificity | 100% | 100% | without cross-reacting with influenza A virus, influenza B virus, HCV and HEV. | without cross-reacting with influenza A virus, influenza B virus, HCV and HEV. |
| Cost | Moderate | Low | High | Moderate |
| Testing equipment | Fluorescent flashlight, warm water | Warm water | Warm water | Fluorescence detection instruments |
| On-site detection | Yes | Yes | Yes | Not suitable |
| Batch testing | Yes | Yes | Not suitable | Yes |
| Visual detection | Yes | Yes | Yes | No |


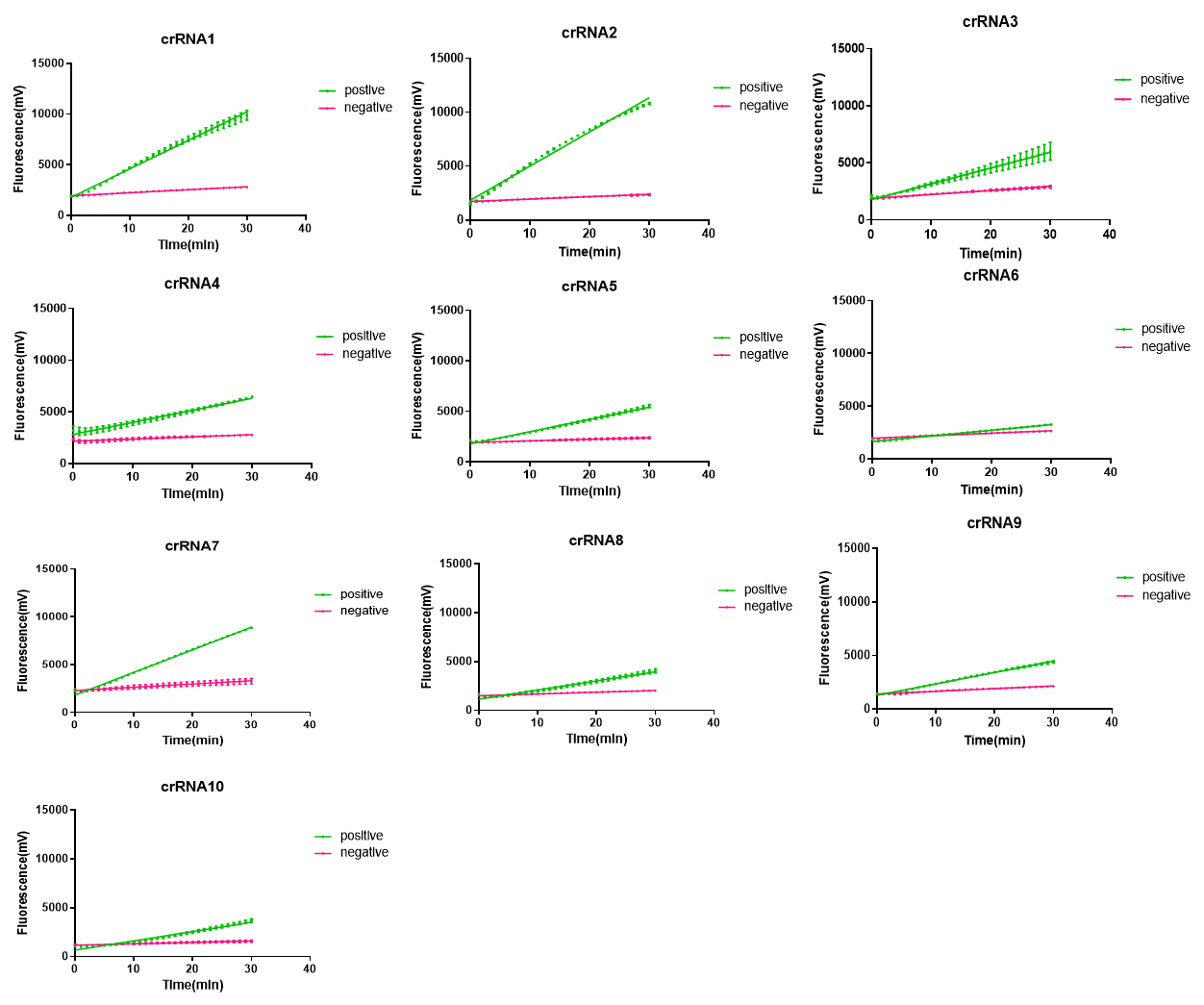
**FIGURE S1 crRNA screening in CRISPR experiments.** Each crRNA was tested individually using the positive or negative plasmids as templates in the initial CRISPR/Cas12a system and the crRNA with the highest slope ratio between the positive group and negative control was considered the optimal one exhibitting the highest reaction efficiency.


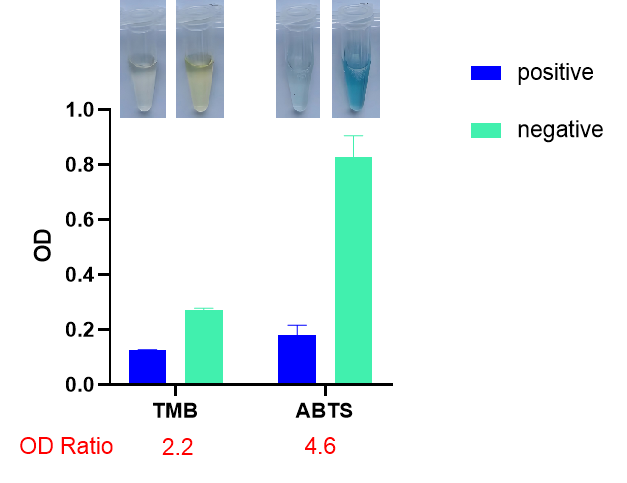


**FIGURE S2 Comparison of the detection performances using TMB and ABTS as substrates in the CRISPR-G4 system.** The optical density at 450nm was measured using TMB, whereas the optical density at 405nm was determined using ABTS. OD Ratio= absorption value of negative group at 405 nm / absorption value of positive group at 405 nm.
